# Dietary supplement beliefs and use patterns among Irish patients with early-stage breast cancer

**DOI:** 10.1007/s11845-024-03676-8

**Published:** 2024-04-06

**Authors:** Eimear O’Donovan, Maeve A. Hennessy, Seamus O’Reilly

**Affiliations:** 1 Dept. of Medical Oncology, Cork University Hospital, Cork, Ireland; 2 Cancer Research @UCC, Cork, Ireland

**Keywords:** Breast cancer, Dietary supplements, Nutrition, Survivorship

## Abstract

**Backround:**

Level one evidence reported poorer outcomes among patients taking dietary supplements after breast cancer (BC) diagnosis.

**Methods:**

We evaluated dietary supplement behaviours among adult BC patients via questionnaire. Sociodemographic data, supplement use, attitudes, and healthcare provider (HCP) advice were analysed.

**Results:**

Of 185 participants, 45% were regular supplement users following diagnosis. Regular supplement use was associated with higher education level (*p* = 0.05). The majority perceived supplements to be safe. Over half reported not receiving advice from HCPs.

**Conclusion:**

In summary, supplement use is prevalent among BC patients. Development of guidelines in relation to safe use of dietary supplements after cancer diagnosis is crucial.

Dietary supplement use has become increasingly popular among breast cancer patients [1]. Recent research has raised safety concerns suggesting the use of antioxidants such as vitamins A, C, and E; carotenoids and coenzyme Q10; and vitamin B12 supplements may be associated with increased recurrence and mortality rates in patients with early breast cancer [2]. It has been postulated that the antioxidant properties of certain supplements may interfere with the oxidising free radicals of chemotherapy and radiotherapy, reducing their ability to target cancer cells [3]. Conversely, several publications indicate that dietary supplement use may play a role in optimising cancer treatments and improving breast cancer outcomes [4]. The contradictory findings to date, in combination with data largely drawn from observational cohort studies, limit their validity. Consequently, this study aimed to characterise dietary supplement behaviours, perceptions, and attitudes among breast cancer patients and to assess the prevalence of supplement use both pre- and post-breast cancer diagnosis. 

This was a multicentre study conducted between August, 2021, and November, 2022, in adult patients diagnosed with early stage breast cancer at Cork University Hospital. An anonymous patient administered questionnaire was devised by the authors and circulated to those attending clinics. The study was approved by the institutional ethics committee and all participants provided informed consent. Behaviours, attitudes, and perceptions regarding supplements, as well as sociodemographic and lifestyle data were recorded. Supplement use patterns both pre- and post-breast cancer diagnosis were collected. Regular use was defined as greater than 3 different dietary supplements taken at least once weekly, generating two distinct groups. Data analysis was conducted using IBM SPSS V28.0.0.0. Demographic characteristics associated with regular use were analysed via logistic regression. Multiple chi-squared tests were performed to determine the association between post-diagnosis regular use and non-regular use and survivors’ attitudes and perceptions, and healthcare provider advice. Statistical significance was assumed by a *p*-value < 0.05.

A total of 185 questionnaires were completed, with baseline demographics shown in Table 1. Regular supplement use following breast cancer diagnosis was reported by 45% of participants, with 16% being new regular users. Regular use prior to diagnosis was associated with increased age, higher education levels, and lower BMI (*p* < 0.05), while regular use following diagnosis was associated with higher education level (*p* = 0.05). The most commonly used dietary supplements were multivitamins, vitamin C, vitamin D, calcium, and omega 3 (Fig. 1). The use of calcium and vitamin D was considerably increased following diagnosis.

**Table 1:** Summary of participant characteristics

Socioeconomic and lifestyle demographic characteristics		No. of participants	%	Mean	Range
Age				57	53
Sex	Male	2	1.1%		
	Female	183	98.9%		
BMI				27.3	25.6
Education level	Primary school	8	4.3%		
	Secondary school	103	56.0%		
	Third level education—bachelor	46	25.0%		
	Third level education—master	27	14.7%		
Exercise/physical activity	More than 4 times a week	73	39.5%		
	3–4 times a week	68	36.8%		
	1–2 times a week	33	17.8%		
	Less than once a week	11	5.9%		
Daily fruit and vegetable	> 5	24	13.0%		
intake	4–5	73	39.5%		
	2–3	77	41.6%		
	1	11	5.9%		
	None	0	0.0%		
Weekly alcohol intake	> 14 units	3	1.6%		
	10–14 units	13	7.0%		
	5–9 units	35	18.9%		
	1–4 units	57	30.8%		
	None	77	41.6%		
Smoking status	Current smoker	14	7.6%		
	Ex-smoker	67	36.2%		
	Never smoker	104	56.2%		
Hormone therapy		128	69.6%		
Chemotherapy		130	70.7%		
Surgery		149	81.0%		
Radiation therapy		143	77.7%

**Fig. 1:**
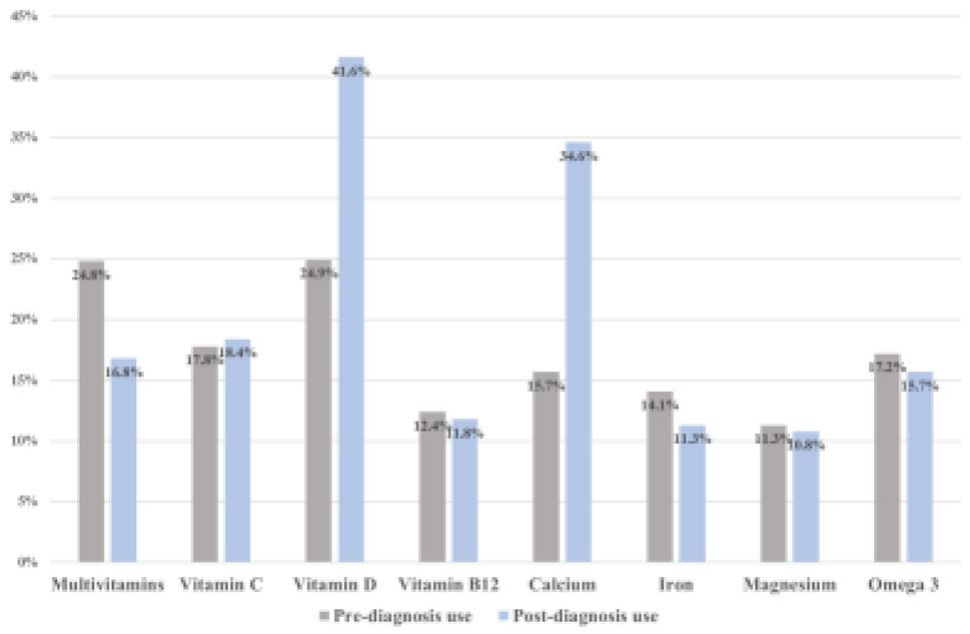
Most commonly used dietary supplements and proportion of use by breast cancer patients pre- and post-breast cancer diagnosis

The majority of patients (77%) desired more information regarding safe dietary supplement use in survivorship. Many were uncertain as to whether dietary supplement intake affected their risk of breast cancer development, recurrence and mortality, and about the safety of supplements when used concurrently with cancer treatments (Fig. 2). These participants were more likely to be non-regular users. A significant proportion believed dietary supplement use had no effect on breast cancer outcomes, a belief evenly distributed between groups. Those who perceived supplement use carried a reduced risk of poorer outcomes were more likely to be regular users. Very few participants felt supplement use increased the risk of poor outcomes (Table 2).

**Fig. 2:**
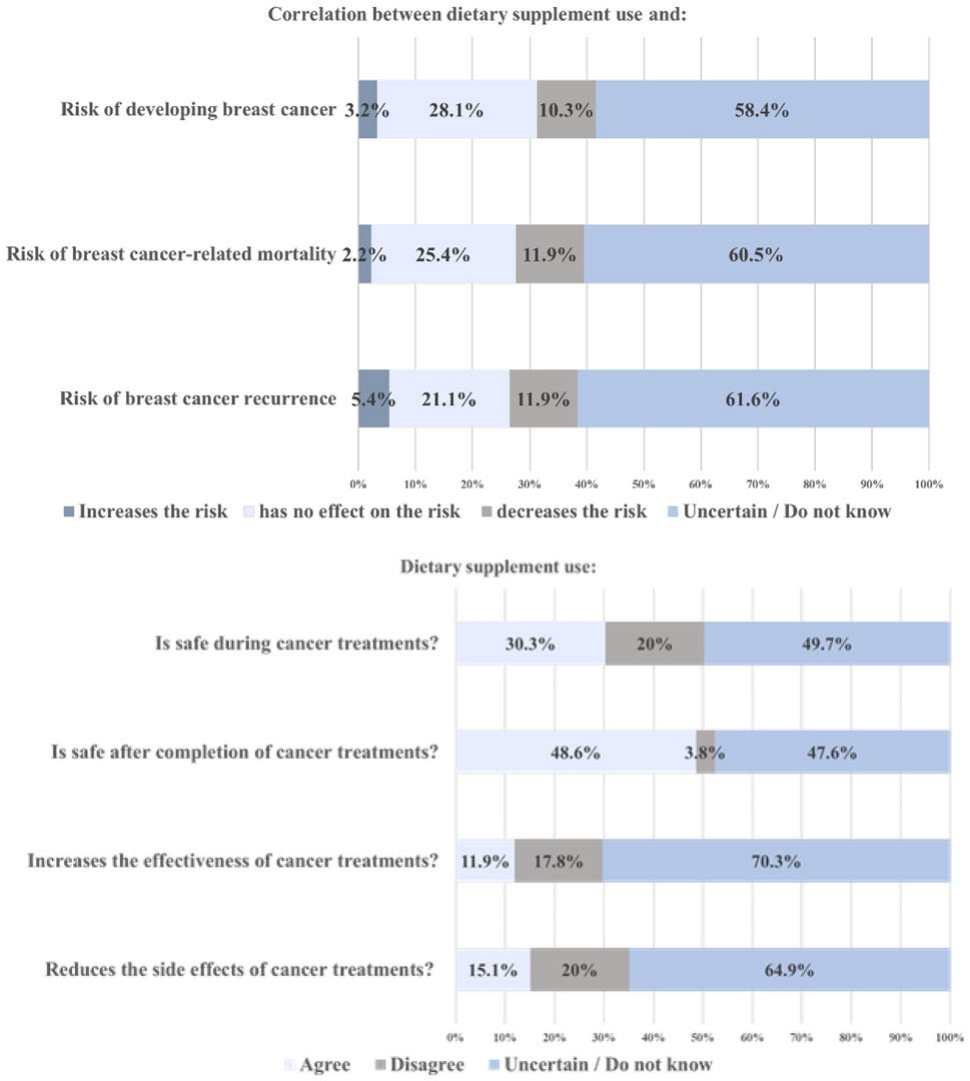
Attitudes toward and perceptions of dietary supplement use in breast cancer patients

**Table 2:** Association of post-diagnosis regular and non-regular supplement use with attitudes and perceptions

Attitudes & Perceptions		Post diagnosis regular users *N* (%)	Post diagnosis non-regular users *N* (%)	*p*-value	95% CI
Dietary supplement use and risk of developing breast cancer	Increases risk	1 (0.5)	5 (2.7)	0.016	0.013-0.018
	No effect on risk	26 (14.1)	26 (14.1)		
	Decreases risk	14 (7.6)	5 (2.7)		
	Uncertain/Do not know	43 (23.2)	65 (35.1)		
Dietary supplement use and risk of breast cancer recurrence	Increases risk	1 (0.5)	9 (4.9)	0.000	0.000<0.001
	No effect on risk	20 (10.8)	19 (10.3)		
	Decreases risk	18 (9.7)	4 (2.2)		
	Uncertain/Do not know	45 (24.3)	69 (37.3)		
Dietary supplement use and risk of breast cancer-related mortality	Increases risk	0 (0)	4 (2.2)	0.014	0.012-0.017
	No effect on risk	26 (14.1)	21 (11.4)		
	Decreases risk	14 (7.6)	8 (4.3)		
	Uncertain/Do not know	44 (23.8)	68 (36.8)		
Dietary supplement use is safe during cancer treatments	Agree	33 (17.8)	23 (12.4)	0.033	0.029-0.037
	Disagree	12 (6.5)	25 (13.5)		
	Uncertain/Do not know	39 (21.1)	53 (28.6)		
Dietary supplement use is safe after the completion of cancer treatments	Agree	47 (25.4)	43 (23.2)	0.186	0.178-0.193
	Disagree	3 (1.6)	4 (2.2)		
	Uncertain/Do not know	34 (18.4)	54 (29.2)		
Dietary supplements increase the effectiveness of cancer treatments	Agree	15 (8.1)	7 (3.8)	0.052	0.047-0.056
	Disagree	12 (6.5)	21 (11.4)		
	Uncertain/Do not know	57 (30.8)	73 (39.5)		
Dietary supplements reduce the side effects of cancer treatments	Agree	16 (8.6)	12 (6.5)	0.314	0.305-0.323
	Disagree	18 (9.7)	19 (10.3)		
	Uncertain/Do not know	50 (27)	70 (37.8)		
It is important to inform healthcare providers about the dietary supplements I am taking	Agree	68 (36.8)	87 (47)	0.421	0.411-0.430
	Disagree	1 (0.5)	0 (0)		
	Uncertain/Do not know	15 (8.1)	14 (7.6)		
I would like more information on dietary supplements	Agree	68 (36.8)	72 (38.9)	0.176	-
	Disagree	15 (8.1)	26 (14.1)		

*CI* confidence interval

Over half of the study participants (54%) received no recommendations or advice regarding dietary supplements from healthcare providers, 21% were recommended to use dietary supplements, while the remainder were recommended to avoid supplement use.

Our findings highlight that while many patients believe dietary supplements are worthwhile, the majority of study participants did not receive advice regarding supplement use from healthcare providers. Dangerous misconceptions regarding ‘cancer-beating diets’ are widespread on social media and potentially harmful for patients with cancer especially those with lower health literacy, reinforcing the need for robust evidence-based information [5].

The most commonly used supplements in this study were multivitamins, vitamin C, vitamin D, calcium, and omega 3. The increase in vitamin D and calcium use may reflect guidelines supporting adjuvant bisphosphonates with calcium and vitamin D to improve outcomes and alleviate the side effects of treatments [6]. Other supplements commonly used by breast cancer patients to mitigate side effects of therapy include vitamin E for taxane-induced peripheral neuropathy, glutamine for mucositis, and selenium for post-operative upper limb lymphoedema [7]. Large randomised trials have previously demonstrated increased risk of lung and gastric cancer with beta-carotene use, as well as an association between vitamin E and prostate cancer, with mixed effects of antioxidants on chemotherapy toxicity [8]. Unmonitored use of herbal medicines is becoming a more frequent phenomenon [9]. Additionally in the era of immunotherapy, the evolving role of the gut microbiome and interactions between what we ingest and treatment outcomes will be a major research area [10]. Taking all of the above into consideration, open communication between patient and physician regarding dietary supplement use is critical.

The strengths of this study include a substantial data collection window,, as well as broad selection criterion, with no exclusion of particular demographic characteristics, current disease activity, or co-morbidities, generating a study sample more representative of the ‘real-world’. Limitations include the self-reporting of supplement use, particularly pre-diagnosis use, creating the potential for recall bias. Our initial ambition was to perform a survey of healthcare professional attitudes in parallel with this study, in order to assess healthcare provider advice concerning dietary supplements in breast cancer survivorship; however, unfortunately we did not have adequate response rates for this to be feasible.

In conclusion, dietary supplement use is prevalent among breast cancer patients; however, attitudes and perceptions suggest a lack of awareness of potential consequences of dietary supplements in breast cancer survivorship. Patients also report a lack of sufficient guidance from healthcare providers regarding dietary supplement use. Future steps include exploration of healthcare provider attitudes and knowledge of dietary supplements and recommendations. Large-scale randomized controlled trials (RCTs) to compile safety data on individual dietary supplements in order to provide robust evidence would be optimal to guide clinical practice. Recommendations for nutritional supplements and dietary advice should be integrated into oncology patient care plans and survivorship guidelines.

## Data Availability

Data from the study available via corresponding author.
